# Knowledge Domain and Hotspots Predict Concerning Electroactive Biomaterials Applied in Tissue Engineering: A Bibliometric and Visualized Analysis From 2011 to 2021

**DOI:** 10.3389/fbioe.2022.904629

**Published:** 2022-05-23

**Authors:** Wentao Xiong, Sheng Wang, Ziheng Wei, Yibo Cai, Bo Li, Feng Lin, Demeng Xia

**Affiliations:** ^1^ Department of Orthopedic, Hainan Hospital of Chinese PLA General Hospital, Sanya, China; ^2^ Department of Emergency, Changhai Hospital, Naval Medical University, Shanghai, China; ^3^ Department of Orthopedics, Shanghai General Hospital Affiliated to Shanghai Jiaotong University, Shanghai, China; ^4^ Department of Orthopedics, Changhai Hospital, Naval Medical University, Shanghai, China; ^5^ Luodian Clinical Drug Research Center, Shanghai Baoshan Luodian Hospital, Shanghai University, Shanghai, China

**Keywords:** electroactive biomaterials, tissue engineering, bibliometrics, hotspot, web of science

## Abstract

**Objective:** Electroactive biomaterials used in tissue engineering have been extensively studied. Electroactive biomaterials have unique potential advantages in cell culture and tissue regeneration, which have attracted the attention of medical researchers worldwide. Therefore, it is important to understand the global scientific output regarding this topic. An analysis of publications on electroactive biomaterials used in tissue engineering over the past decade was performed, and the results were summarised to track the current hotspots and highlight future directions.

**Methods:** Globally relevant publications on electroactive biomaterials used in tissue engineering between 2011 and 2021 were extracted from the Web of Science database. The VOSviewer software and CiteSpace were employed to visualise and predict trends in research on the topic.

**Results:** A total of 3,374 publications were screened. China contributed the largest number of publications (995) and citations (1581.95, actual value ×0.05). The United States achieved the highest H-index (440 actual values ×0.05). The journal Materials Science & Engineering C-materials for Biological Applications (IF = 7.328) published the most studies on this topic (150). The Chinese Academy of Science had the largest number of publications (107) among all institutions. The publication titled Nanotechnological strategies for engineering complex tissues by Dir, T of the United States had the highest citation frequency (985 times). Regarding the function of electroactive materials, the keyword “sensors” emerged in recent years. Regarding the characterisation of electroactive materials, the keyword “water contact angle” appeared lately. Regarding electroactive materials in nerve and cardiac tissue engineering, the keywords “silk fibroin and conductive hydrogel” appeared recently. Regarding the application of electroactive materials in bone tissue engineering, the keyword “angiogenesis” emerged in recent years. The current research trend indicates that although new functional materials are constantly being developed, attention should also be paid to their application and transformation in tissue engineering.

**Conclusion:** The number of publications on electroactive biomaterials used in tissue engineering is expected to increase in the future. Topics like sensors, water contact angle, angiogenesis, silk fibroin, and conductive hydrogels are expected to be the focuses of research in the future; attention should also be paid to the application and transformation of electroactive materials, particularly bone tissue engineering. Moreover, further development of the field requires joint efforts from all disciplines.

## Introduction

The loss or failure of an organ or tissue is one of the most frequent, devastating, and costly problems in human health (Langer and Vacanti, 1993). Current treatments include organ transplantation, surgical reconstruction, mechanical devices, or metabolite supplementation (Lalan et al., 2001). While these strategies represent significant advances in the field of medicine, some inherent limitations are unavoidable, such as tissue transplantation being limited by the nature of the material, which often brings new trauma or secondary injury to patients. In addition, tissue transplantation is often limited by a lack of donors and has risks like disease transmission and immune rejection.

Artificial bioengineered tissue repair materials, with excellent biocompatibility and bioactivity, created *via* tissue engineering (TE) have gradually become a promising strategy in the clinical treatment of tissue defect repair in recent years. In addition, they have the advantages of ubiquitous sources, easy preparation, and safety. In TE the principles and methods of engineering and the life sciences are applied for developing biological substitutes for restoring, maintaining, or improving functions (Nerem, 1992). It is one of the most relevant topics within the field of advanced therapies (Santisteban-Espejo et al., 2018), and has a wide range of potential applications in tissue repair and regeneration.(Naveau et al., 2017). TE has been widely used in the regeneration of skin (Debels et al., 2015), bone (Pati et al., 2015), cartilage (Ren et al., 2015), nerve tissue (Georgiou et al., 2015), and blood vessels (Jaspan and Hines, 2015). Undoubtedly, TE is becoming a novel approach to future therapeutic applications in the clinical treatment of tissue defect repair.

The rapid development of bioscaffolds in tissue engineering over the past few decades has been fuelled by rising standards for effective bioscaffolds, which play an important role in the tissue repair process. A new generation of smart bioscaffolds not only serves as a medium or matrix for cell adhesion but also modulates cells, supports the process of cell proliferation, and promotes new tissue specialisation (Sukmana, 2012; Guo and Ma, 2018). Inspired by studies on the electrophysiological behaviour of cells and tissues, electroactive biomaterials have been proposed for and applied in tissue engineering and regenerative medicine research.

**FIGURE 1:**
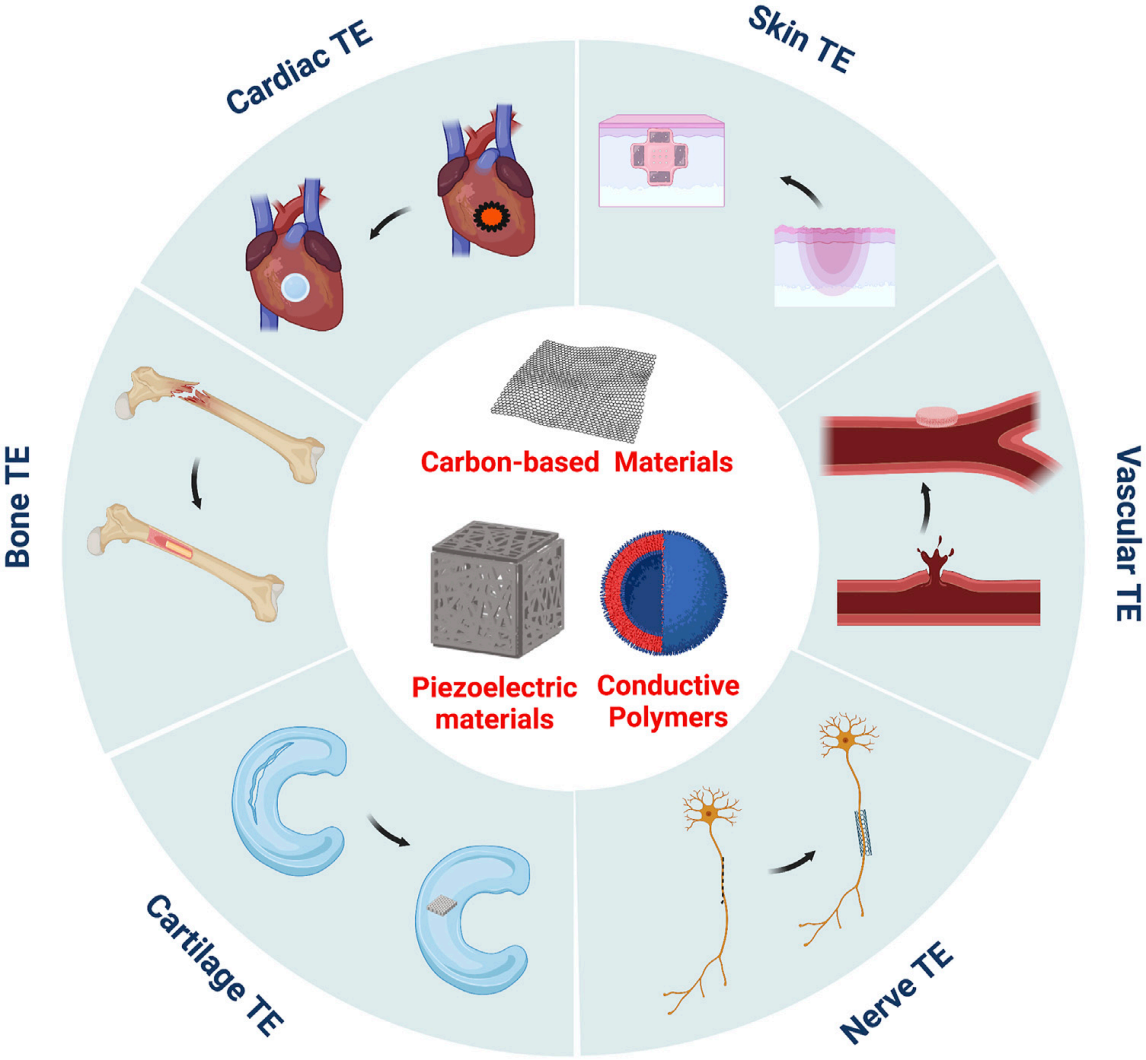
Electroactive biomaterials have been widely used in the TE of skin, bone, cartilage, nerve tissue, cardiac or blood vessels.

Electroactive biomaterials originated from the first measurements of the piezoelectric effect of bone tissue in 1957 by (Brown, 1980). Subsequently, research on the role of the piezoelectric effect in regulating cell behaviour and controlling the growth and remodelling of bone tissue has become more and more extensive, which has led to a series of piezoelectric materials being proposed and applied in the field of biomedical research. In addition to piezoelectric materials, researchers developed conductive polymers and carbon-based nanomaterials. Conductive polymers can concentrate externally applied electrical stimuli in their surrounding areas by regulating the loaded stimuli spatially (Guarino et al., 2013), promoting intracellular DNA synthesis, and accelerating cell division and proliferation (Stewart et al., 2015). Carbon-based nanomaterials, such as graphene sheets and carbon nanotubes, possess unique mechanical, electrical, and optical properties, have good biocompatibility at a certain concentration, and can support cell adhesion, proliferation, and differentiation. They present new opportunities for tissue engineering and are potential candidates for the development of artificial scaffolds (Ku et al., 2013). In conclusion, electroactive biomaterials can be combined with a human bioelectric system to directly transmit electrical, electrochemical, and electromechanical signals to cells and induce cell differentiation and tissue regeneration. Therefore, these materials have unique potential advantages in cell culture and tissue regeneration (Guimard et al., 2007) (Figure 1), which has attracted the attention of medical researchers worldwide.

Recently, many reviews have highlighted the current strategies and future prospects of electroactive biomaterials in TE; however, researchers may merely explore the literature in a single direction, which may lead to narrow-minded exploration. Moreover, each article summarises different keywords, and a large number of keywords make it difficult to accurately locate information in the PubMed, Web of Science (WOS), and Scopus databases. Hence, comprehensively analysing the status of this topic and revealing the current or future hotspots from multiple perspectives are urgent requirements.

Bibliometrics is a statistical and quantitative method used to analyse the academic influence and characteristics of scientific output. Combined with creative design and information visualisation, bibliometric mapping can visually represent bibliometric data (Zou et al., 2018). We performed a bibliometric analysis using international databases to identify hotspots in previous reports, such as research progress on heat stroke (Xia D. M et al., 2021) and hotspots concerning the use of stem cells for cartilage regeneration (Xia D et al., 2021). In bibliometrics, co-occurrence analysis is used to define research hotspots. We consider that if two terms appear in the same article concurrently, they may have a potential relationship. Furthermore, if these two terms appear frequently at the same time in the same article, they are considered to be closely related. After some analysis of these co-occurrence relations, such as cluster analysis or factor analysis, keywords that reach a threshold are considered to represent hot topics in the research area (Li et al., 2015). In addition, bibliometrics have been widely used in the fields of information science, chemistry, and physics, and show potential in the field of medicine (van Eck and Waltman, 2010). Using bibliometrics, researchers can determine more specific research themes, thereby achieving a more comprehensive understanding of the relationships between specific research areas.

We applied a bibliometric analysis to uncover global research trends, evaluate achievements related to electroactive biomaterials used in TE and predict possible future hotspots. As expected, the data extracted from this analysis could indicate the most productive areas in the evolution of electroactive biomaterials with the goal of facilitating the clinical translation of electroactive biomaterials into tissue engineering and providing references for future developments.

## Materials and Methods

### Data Sources and Search Strategies

**FIGURE 2:**
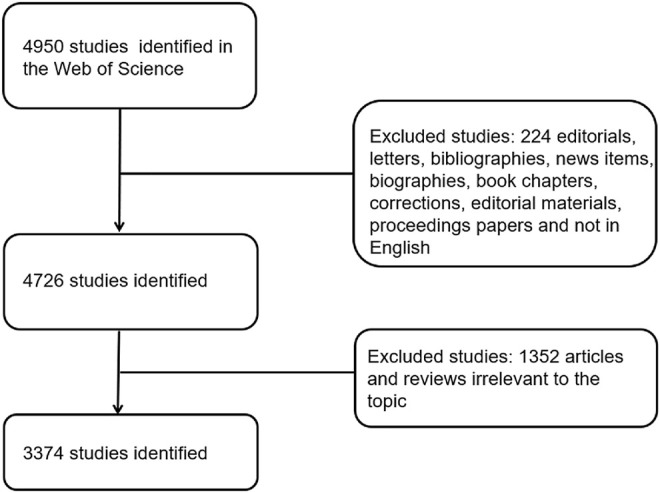
Flow diagram of the inclusion process. The detailed process of screening and enrolment (irrelevant articles were manually screened by two authors through abstracts and full texts), and articles irrelevant to the topic were excluded.

Web of Science (WOS), a database containing a large amount of physical, biological, and medical information, has often been used in bibliometric research. In our research, we performed a search for studies relevant to electroactive biomaterials used in tissue engineering (TE) between January 2011 and December 2021. All searches were conducted on 17 February 2022 to avoid bias according to database renewal. The search strategy was as follows: TS = [(electroactive, conductive, piezoelectric, or carbon-based) and (material or biomaterial, hydrogel, scaffolds, polymers, or ceramics) or polypyrrole or PPy, polyaniline, PANi, aniline oligomer, polyvinylidene fluoride, PVDF, Kynoar, l-polylactic acid, polylactic-L acid, PLLA, graphene, carbon nanotubes, or CNT] and (tissue engineer* or regenerat* medicine). The types of studies we included were strictly screened; only original articles or reviews were included in our analysis, and all other types of studies were excluded. Finally, articles irrelevant to the topic were filtered manually. The process associated with research screening was undertaken by two authors (WTX and SW); if there was disagreement during the screening process, it was up to an experienced corresponding author to decide whether to include that paper in our study. Detailed information on enrolment and selection is shown in **(** Figure 2 **).**

### Data Collection and Processing

Basic information on the research in terms of titles, keywords, authors, publication dates, countries and regions of origin, institutions, journals, and overall information about the article in terms of number of citations, H-index, etc. were extracted from the identified publications by three authors (WTX, SW, and DMX). Several data analysis tools were used for data analysis and processing. Microsoft Excel 2016, GraphPad Prism 8, VOSviewer version 1.6.12, and CiteSpace version 5.6. R5 64 and an online analysis platform (http://bibliometric.com/) were applied to present, analyse, and describe the data.

### Bibliometric Analysis

WOS, as a database covering a large amount research on medical, physical, and materials sciences, has a wide-ranging and comprehensive content; therefore, WOS was chosen as our preferred database for bibliometric research. The three most important indicators of article quality evaluation include the impact factor (IF), H-index, and relative research interest (RRI), and these were the objects of our research. The impact factor (IF) was taken from the Journal Citation Reports (JCRs), which is recognised as a key indicator in evaluating articles (Kavic and Satava, 2021). In general, the impact factor can directly reflect the quality and influence of an article. The H-index of an article, as a measure of academic productivity, indicates that a researcher or country has published at least H papers on a particular topic, that each paper has been cited at least H times. The indicator is objective in assessing the quality of the article (Favre et al., 2020). The relative research interest (RRI) is related to the number of publications in the field and the total number of publications included in the WOS database, which is meaningful for evaluating the popularity of research in the field (Wang et al., 2019). VOSviewer, a practical statistical software, can use text downloaded by WOS to perform a visual analysis of the references, institutions, authors, and terms. This software was used to display the time distribution and dynamic variability of keywords, and accurately reveals the evolution trend of hotspots in the research field (Chen et al., 2021). CiteSpace, which uses the Java programming language, is a useful tool for data analysis and processing. In addition to conventional analysis, an analysis of the cooperative relationships between related fields is unique in this research field (Yao et al., 2020).

## Results

### Field Activity Analysis

**FIGURE 3:**
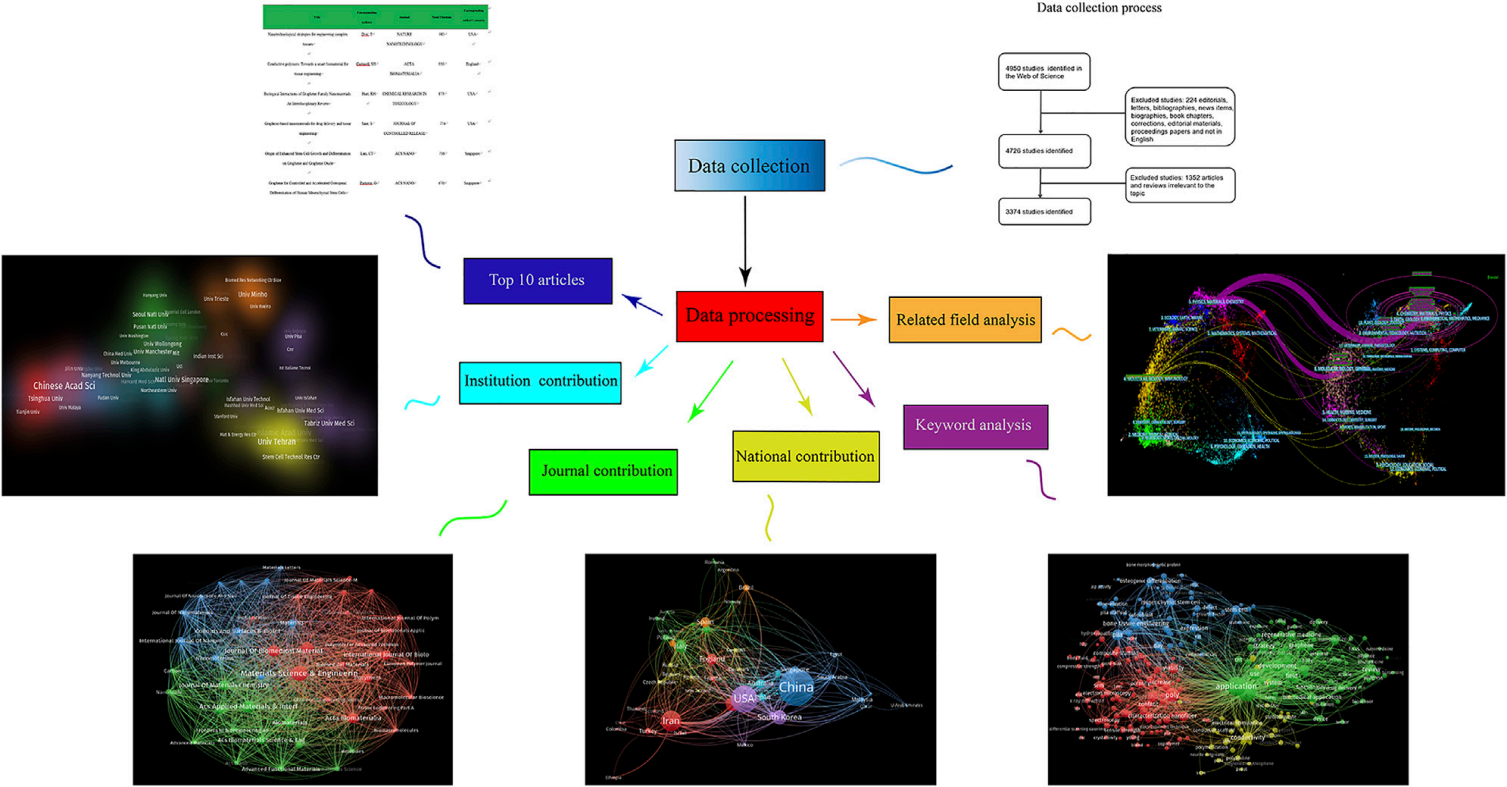
Flow diagram of six aspects of electroactive biomaterials used in tissue engineering. Contributions of countries, contributions of different journals and top 10 articles, contributions of different institutions, keywords, and related fields are listed.

According to the inclusion criteria, 3,374 articles related to electroactive biomaterials used in TE were included in the final analysis. By analysing and summarising the data, six aspects of the contributions, i.e., countries, contributions of different journals, top 10 articles, contributions of different institutions, keywords, and related fields were presented in the results. The specific process is shown in Figure 3.

### Global Contribution to the Field

**FIGURE 4:**
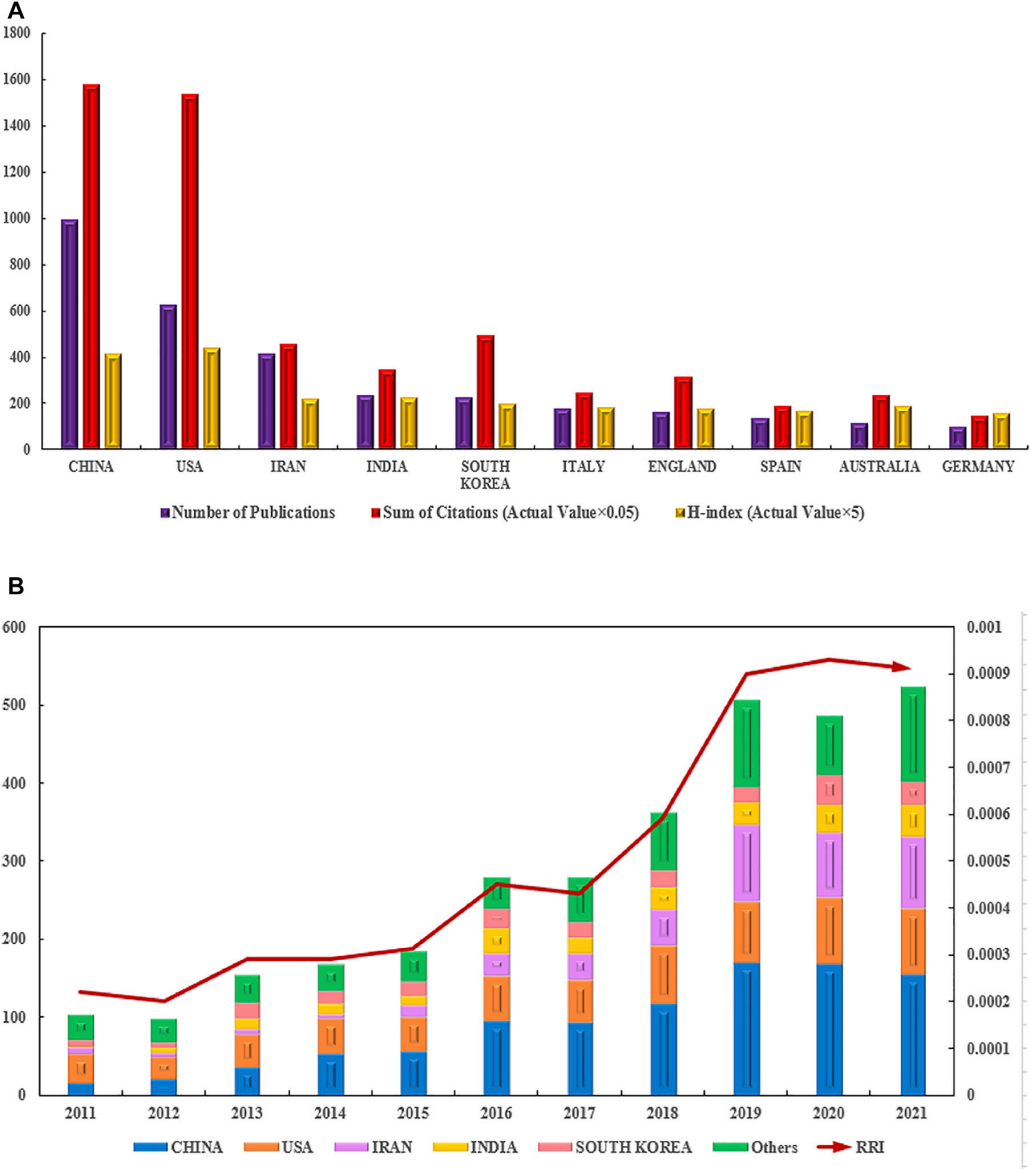
Contributions of different countries/regions to the research field regarding electroactive biomaterials used in TE. **(A)** The number of publications, citation frequency (× 0.05) and H-index (× 5) in the top 10 countries or regions; **(B)** The number of publications worldwide and the timings of the relative research interests of electroactive biomaterials used in TE. (RRI = Relative research interest).

According to the national distribution of publications, China (995 publications) was the most productive country, followed by the United States (626 publications), Iran (417 publications), India (236 publications), and South Korea (228 publications) (Figure 4A). The top 10 countries with the most publications are shown in Figure 4.

In terms of total citations, the top five countries/regions are China with 31,639, the United States with 30,806, Iran with 9146, India with 6,995, and South Korea with 9862, respectively. The countries/regions with the highest H-index are as follows: the United States 88, China 83, India 45, Iran 44, and South Korea 40 (Figure 4A). According to the annual distribution of publications, although there was a slight decline during 2011–2012, the overall trend in publication output increased from 2011 to 2021 (100–500 publications per year). The trend of RRI was similar, suggesting that the field of electroactive biomaterials used in TE is receiving more attention in general (Figure 4B). It can be predicted that the growth trend in the number of publications in this field will accelerate in the future.

**FIGURE 5:**
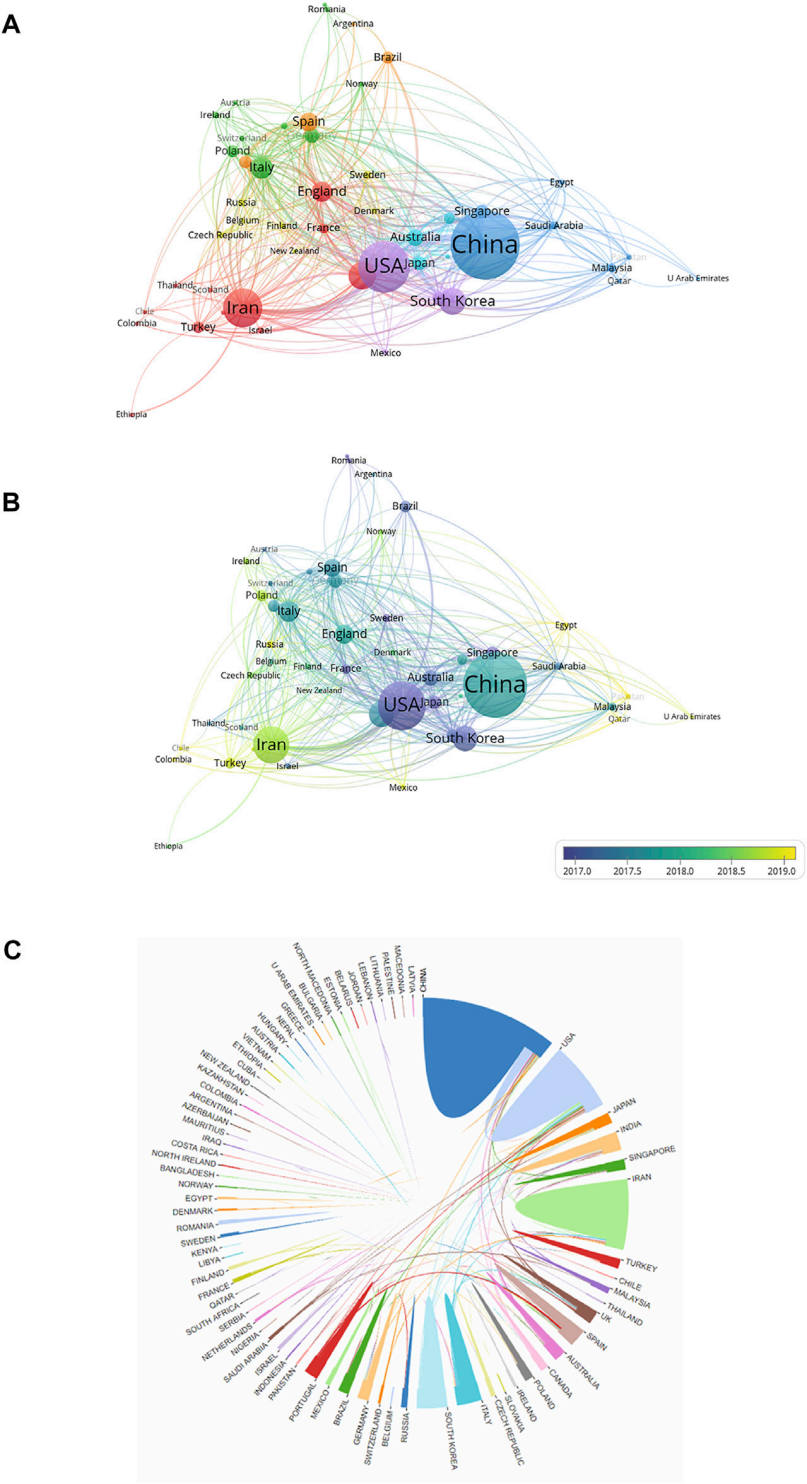
Cooperation network of countries/regions with regard to research on electroactive biomaterials used in TE. **(A)** The network of cooperative relations between countries/regions was established with VOSviewer; **(B)** Concentrated years of publications for different countries; **(C)** The cooperative relations between countries/regions were visualized.

Collaborations between countries/regions are shown in Figure 5. The size of the circles indicates the number of publications and the width of the connecting line between the two circles indicates the degree of collaboration (Figure 5A). Many countries/regions have some years with a concentrated article output. The United States and South Korea had the most publications before 2017. China, Italy, and England intensively published articles in 2018. Articles from Iran were published mainly after 2018 (Figure 5B). As the top two countries with the most publications, the United States and China cooperated the most in this field, and the United States had the strongest total link strength, which means that the United States had the predominant influence in this field **(** Figure 4C **)**.

### Analysis of Journal Distribution

**FIGURE 6:**
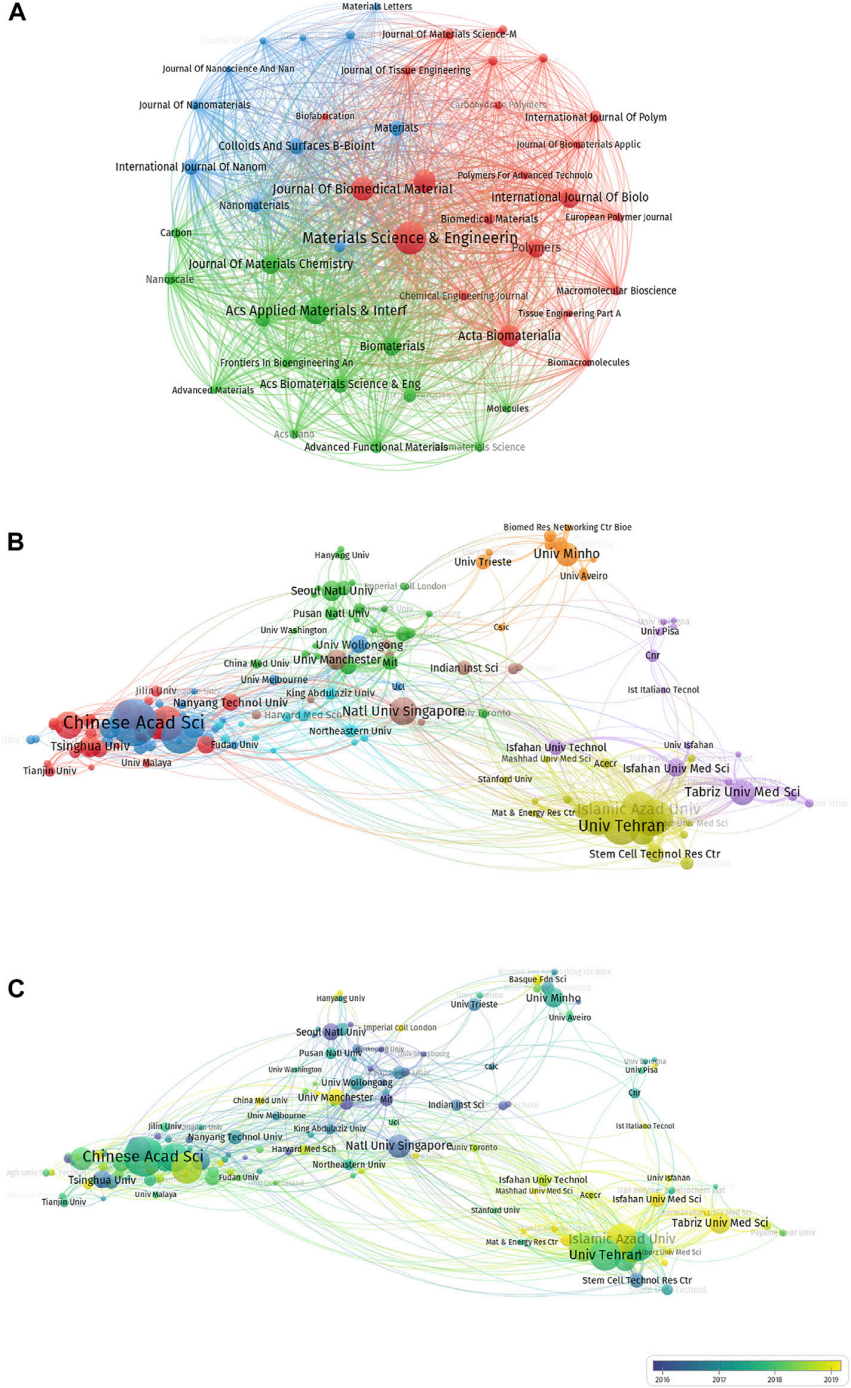
The distribution of journals and institutions engaged in research on electroactive biomaterials used in TE. **(A)** Top 10 journals by number of publications, blue bar representing the proportion and red bar representing IF; **(A)**. The network of relationships between with different journals in VOSviewer. **(B)** The network of institutions produced in VOSviewer. The size of the circles reveals the number of publications; **(C)** Concentrated years of publications for different countries.

Different journals are active in different fields of publication. Therefore, we performed a journal distribution analysis of publications on the use of electroactive biomaterials in TE. The journal Materials Science & Engineering C-materials for Biological Applications (IF = 7.328) published the most studies, with 150 publications. There were 107 articles on electroactive biomaterials used in TE in ACS Applied Materials & Interfaces (IF = 9.229), 91 articles in the Journal of Biomedical Materials Research Part A (IF = 4.396, 2019), and 81 articles in RSC Advances (IF = 3.361). The top 10 journals with the highest number of publications are listed in Table 1. The journals did not just represent themselves; the fields behind them are more worthy of exploration and research. Materials Science and Engineering C-materials for Biological Applications and the Journal of Biomedical Materials Research Part A have the closest relationship. As far as this field is concerned, journals with high volumes of publications also have a high centrality and a focus on tissue engineering and materials science **(** Figure 6B).

**TABLE 1:** The top 10 journals publishing articles on the electroactive biomaterials used in TE.

SCR^a^	Journal	Contribution (%)	IF^b^
1st	Materials science & engineering C-materials for biological applications	4.707	7.328
2nd	ACS applied materials & interfaces	3.357	9.229
3rd	Journal of biomedical materials research Part a	2.855	4.396
4th	Rsc advances	2.542	3.361
5th	Acta biomaterialia	2.447	8.947
6th	International journal of biological macromolecules	2.196	6.953
7th	Polymers	2.102	4.329
8th	Journal of materials chemistry B	2.04	6.331
9th	Biomaterials	1.945	12.48
10th	ACS biomaterials science & engineering	1.883	4.749

SCR, standard competition ranking; IF, impact factor; OA open access.

a Equal journals have the same rank, and then a gap is left in the ranks.

b The impact factor was reported according to journal citation reports (JCR) 2021.

### Analysis of Institution Distribution

Five of the top 10 institutions in this field are in Iran, the other three institutions are in China, and one is in the United States (Table 2). In terms of publication ranking, the first was the Chinese Academy of Sciences (107 publications), followed by the University of Tehran (78 publications), the Shanghai Jiao Tong University (77 publications), Islamic Azad University, and Sichuan University (73 publications). The top 10 institutions with the most publications are shown in Table 2.

**TABLE 2:** Top 10 institutes with the most publications on the electroactive biomaterials used in TE.

Rank	Institution	Contribution (%)	Country
1st	Chinese acad sci	3.36	China
2nd	Univ tehran	2.45	Iran
3rd	Shanghai jiao tong univ	2.42	China
4th	Islamic azad univ	2.29	Iran
4th	Sichuan univ	2.29	China
6th	Amirkabir univ Technol	2.23	Iran
6th	TarbiaT. modares univ	2.23	Iran
8th	Univ tehran med sci	2.04	Iran
9th	Univ michigan	1.85	United states
10th	Xi an jiao tong univ	1.79	China

Figures 6B,C highlight the close and complex collaborative relationships between different institutions. VOS viewer was employed to analyse the centrality of these institutions; the circle indicates centrality, and the area of the circle is proportional to its centrality. Asterisks of the same colour indicate that such organisations belong to the same organisation. The Chinese Academy of Sciences and the University of Tehran are the most prominent institutions, which suggests that they are regarded as pivotal points (Figure 6B). The shade of the colour indicates the time of publication. Although Tarbiat Modares University ranks sixth in terms of the number of articles published, its articles are relatively new, and we predict that this institution will be a centre of research in this area in the future. In Figure 6C, the Chinese institution clusters in the lower left corner and the Iranian institution clusters in the lower right corner are lighter in colour, indicating that these two countries have recently exhibited significant research interest in this field.

### Overview of Landmark Articles and Authors

The top 10 authors with the highest total citations of papers published between 2011 and 2021 are shown in Table 3. As presented in Table 3, there are three from the United States, two from Singapore and China each, and one each from England, Canada, and Iran. Notably, the publication titled Nanotechnological strategies for engineering complex tissues by Dir, T of the United States had the highest citation frequency (985 times). The second-ranked journal and third-ranked journal are Conductive polymers: Towards a smart biomaterial for tissue engineering and Biological interactions of graphene-family nanomaterials: an interdisciplinary review. The content of this article is also the embodiment of the research direction, and nanomaterials have gradually become the focus of our research.

**TABLE 3:** The top 10 authors with the highest total citations of papers published between 2011 and 2021.

Title	Corresponding authors	Journal	Total citations	Corresponding author’s country	Research field
Nanotechnological strategies for engineering complex tissues	Dvir, T	Nature nanotechnology	985	United states	Bone TE. Nerve TE. Cardiac TE
Conductive polymers: Towards a smart biomaterial for tissue engineering	Cartmell, SH	Acta biomaterialia	930	England	Nerve TE
Biological interactions of graphene-family nanomaterials: An interdisciplinary review	Hurt, RH	Chemical research in toxicology	873	United states	Bone TE. Cartilage TE
Graphene-based nanomaterials for drug delivery and tissue engineering	Sant, S	Journal of controlled release	774	United states	Bone TE. Nerve TE
Origin of enhanced stem cell growth and differentiation on graphene and graphene oxide	Lim, CT	ACS nano	736	Singapore	Bone TE
Graphene for controlled and accelerated osteogenic differentiation of human mesenchymal stem cells	Pastorin, G	ACS nano	676	Singapore	Bone TE. Cartilage TE. Skin TE
Carbon-nanotube-embedded hydrogel sheets for engineering cardiac constructs and bioactuators	Tang, XW	ACS nano	567	Canada	Cardiac TE
Size-dependent genotoxicity of graphene nanoplatelets in human stem cells	Akhavan	Biomaterials	516	Iran	Bone TE
Three-dimensional graphene foam as a biocompatible and conductive scaffold for neural stem cells	Dai, JW	Scientific reports	481	China	Nerve TE
Lightweight conductive graphene/thermoplastic polyurethane foams with ultrahigh compressibility for piezoresistive sensing	Liu, CT	Trends in cell biology	470	China	Material preparation

### Co-Occurrence Analysis of Key Words

**FIGURE 7:**
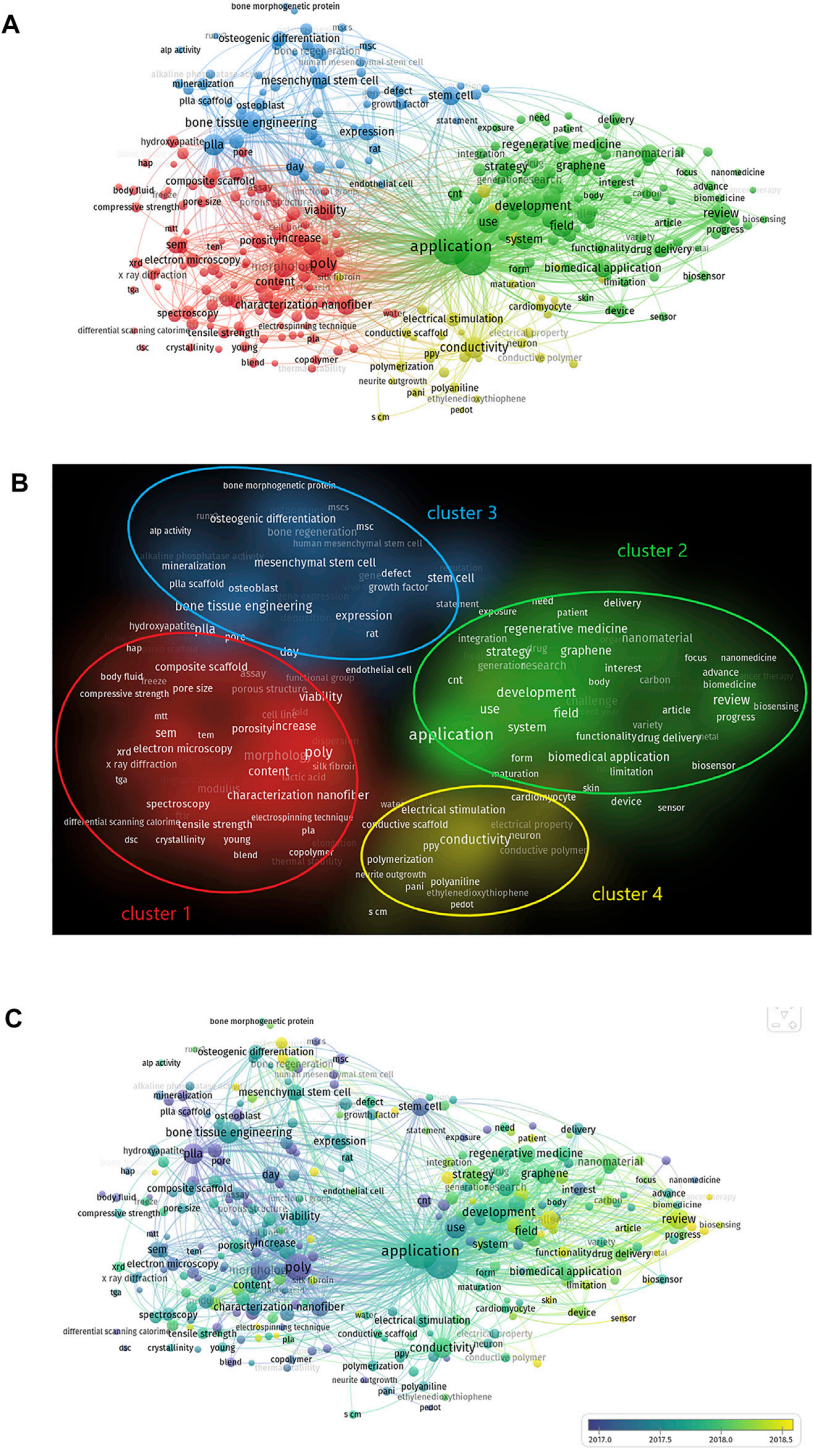
The analysis of keywords in publications on electroactive biomaterials used in TE. **(A)** Mapping of keywords in the field of the use of ultrasound in emergency medicine. The size of the circle represents the frequency with which keywords appear. **(B)** Mapping of the keywords in the area of electroactive biomaterials used in TE. The words were divided into four clusters in accordance with different colours that were generated by default, specifically, characterization of electroactive materials (lower left in red), function of electroactive materials (upper right in green), application of electroactive materials in bone tissue engineering (upper left in blue) and application of electroactive materials in nerve and cardiac tissue engineering (lower right in yellow); **(C)** The distribution of keywords is presented according to the average time of appearance. The blue colour represents an early appearance, and the yellow colour represents a late appearance.

We analysed keywords extracted from 3,374 publications using VOSviewer. As shown in Figure 7A, from a total of 288 keywords, defined as terms that occurred more than 35 times within the titles and abstracts in all papers during the analysis process, the top four keywords that were frequently mentioned are: application (1,513 times), engineering (1,366 times), poly (810 times) and development (539 times). Detailed data on the co-occurrence of all included keywords are presented in Figure 7A.

The keywords were divided into four clusters: characterisation of electroactive materials, function of electroactive materials, applications of electroactive materials in bone tissue engineering, and applications of electroactive materials in nerve and cardiac tissue engineering (Figure 7B). Keywords represent the main topics of publications. Co-occurrence analysis of keywords is conducive to systematically understanding the relationship between keywords, and consequently, grasping the relationship between various topics in this field. Further cluster analysis helps us to systematically understand current progress in this field. VOSviewer was employed to analyse keywords (defined as words that were used more than 35 times in titles and abstracts across all publications) in all included publications. For the characterisation of electroactive materials, the keyword with the highest frequency was poly (810 times). For the function of electroactive materials, this word was application (1,513 times). For the applications of electroactive materials in bone tissue engineering, the word was bone tissue engineering (513 times). For the applications of electroactive materials in nerve and cardiac tissue engineering, the word was conductivity (473 times). (Figures 7A,B).

As shown in Figure 7C, VOSviewer coloured all keywords according to the average number of times the word appeared. Specifically, blue indicates that the word appeared relatively early, while yellow indicates a more recent appearance. Compared with the keywords that appear most frequently, recent keywords can represent current research hotspots, which is of greater interest to us. VOSviewer colours all keywords according to the average number of times the word has appeared. In the cluster of characterisations of electroactive materials, the most recent keyword was water contact angle [42 times, average appearing year (AAY) 2018.3]. In the cluster of functions of electroactive materials, the newest keyword was sensor (82 times, AAY 2018.9). In the cluster of applications of electroactive materials in bone tissue engineering, the newest keyword was angiogenesis (50 times, AAY 2019.14). In the cluster of applications of electroactive materials in nerve and cardiac tissue engineering, the newest keywords were silk fibroin (71 times, AAY 2018.6) and conductive hydrogel (71 times, AAY 2018.3).

### Related Field Analysis

**FIGURE 8:**
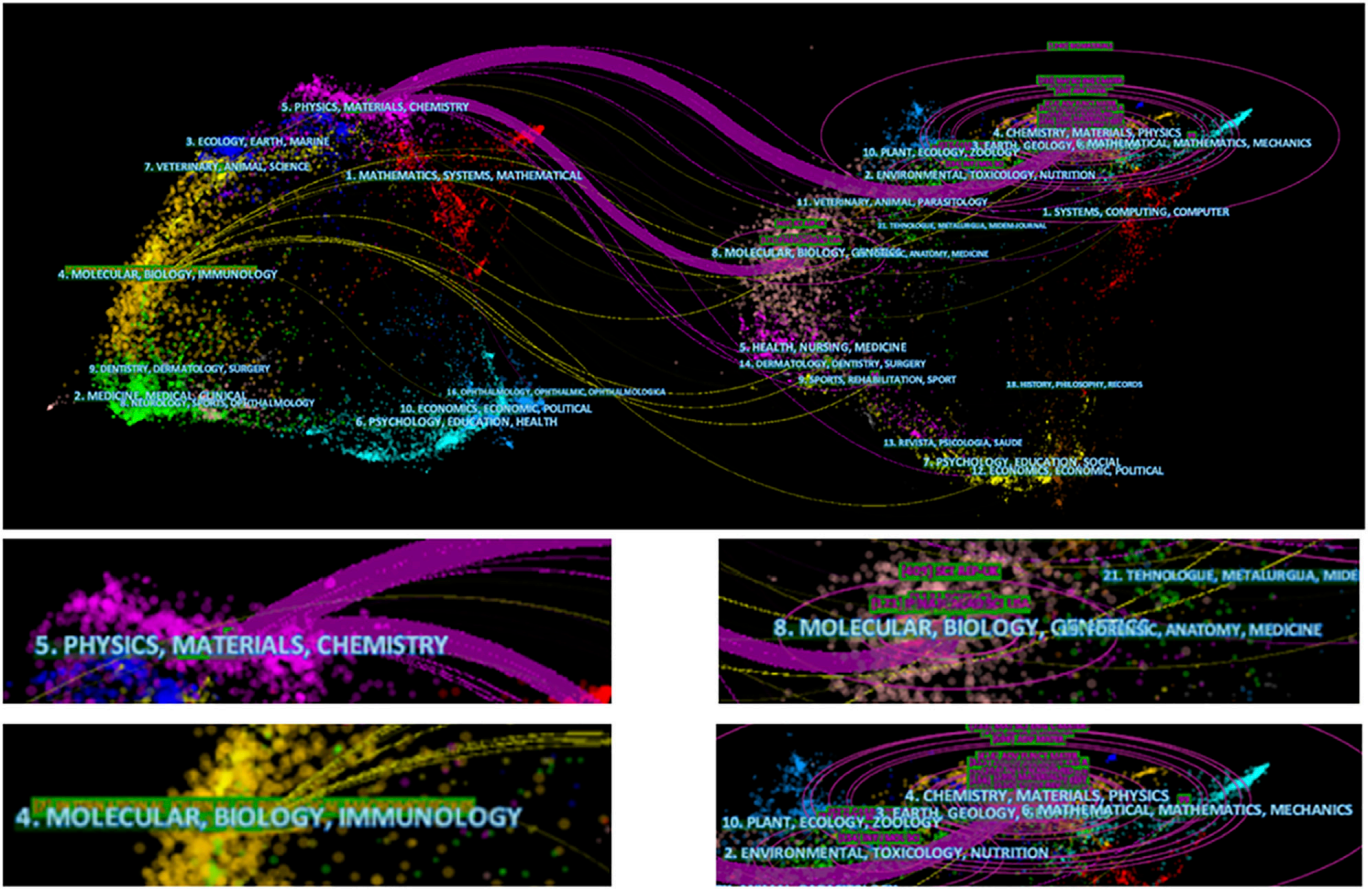
Fields related to electroactive biomaterials used in TE. The lefthand side represents the fields of the articles included in the study, and the righthand side represents the fields of the references of the articles.

In Figure 8, the 3,374 publications included in our research are mainly divided into two fields, one of which includes physics, materials, and chemistry, and the second field, which includes molecular, biological sciences, and immunology. In addition, the references of these 3,374 articles were mainly distributed across the following fields: physics, materials, and chemistry, and molecular, biological, and immunological studies. We found that the use of electroactive biomaterials in TE mainly involved subdisciplines in the fields of physics, materials, chemistry, molecular biology, and immunology. The development of this field is related to a combination of medical and engineering-related disciplines.

## Discussion

### Trends in Research on the Electroactive Biomaterials Used in TE

China contributed the most to the publication volume of all countries (Figure 3A). The proportion of global output is increasing year by year, which shows that China attaches great importance to scientific research in this area (Figure 4B). This is followed by the United States, but publication in the United States has the highest H-index, which shows that United States publication has a greater impact around the world. The country contact map based on WOS data shows that China has connections with many countries which are active in this field, especially with the United States (Figure 4A), and that these two countries have the most publications, which indicates that mutual cooperation plays an important role in advancing the development of this field. Although Iran ranks third in the number of publications, Iran’s publications are mostly after 2018, and their research results are the latest (Figure 4B). With regard to research institutions, the results were also significantly influenced by country; five of the top 10 institutions were from Iran (Table 2), and the latest publications were also concentrated in Iranian institutions (Figure 6C). This shows that Iran’s research in this field is playing an increasingly important role, and that it has emerged as a new research centre.

The top 10 most-cited publications reflect the research hotspots and priorities in the field of electroactive biomaterials used in TE (Table 3). Two of the top three publications are related to nanomaterials, Nanotechnological strategies for engineering complex tissues mainly discusses the impact of nanostructures (Dvir et al., 2011) on the properties of scaffolds and their uses in monitoring the behaviour of engineered tissues. Biological interactions of graphene-family nanomaterials: An interdisciplinary review also proposed a systematic nomenclature for this set of graphene-family nanomaterials (GFNs) and discussed specific material properties relevant in biomolecular and cellular interactions (Sanchez et al., 2012), which indicates that nanomaterials are receiving increasing attention in this field. We also found that seven of the top 10 articles were related to graphene, with papers on the topics of neural stem cells, bone marrow mesenchymal stem cells, platelets, drug transport vectors, and other hot topics. Graphene and its derivatives have many applications in the field of TE owing to their superior properties, such as electrical conductivity, biocompatibility, transparency, high surface area, and superior mechanical strength (Tahriri et al., 2019). In particular, research on genotoxicity and biocompatibility of stem cell regeneration has attracted much attention (Akhavan et al., 2012; Li et al., 2013). We also found that seven were related to bone tissue engineering and four were related to nerve tissue engineering. This shows that the application of electroactive materials in bone and nerve tissue engineering is a research hotspot, and it also reflects the clinical transformation trend of electroactive materials in the future.

In terms of journals, the impact factors were generally high, including in biomedicine and materials (Table 1), which mainly includes a combination of physics, materials, chemistry, molecular biology, and immunology (Figure 8). This suggests that progress in the field requires collaboration across disciplines. Moreover, there has been a steady increase in scholarly interest, as reflected by the rapid increase in the RRI (Figure 4B) in recent years. We believe that there are two main reasons why this has received much attention. On the one hand, with increasing cases of organ shortages and donor scarcity within the last three decades, the research focus in the field of TE continues to advance toward a potential therapy for various types of tissue damage (Sharma et al., 2019; Matai et al., 2020). However, after years of development, an increasing number of electroactive biomaterials are expected to become a new generation of tissue defect repair materials because of their biocompatibility and easily modified surface characteristics, which can convert different types of signals such as mechanical, thermal, and magnetic signals into electrical signals to ultimately regulate tissue regeneration (Finkenstadt and Willett, 2006; Balint et al., 2014).

### Research Focused on Electroactive Biomaterials Used in TE

According to the map based on bibliographic data from the co-occurrence analysis of all keywords (Figures 7A,C), the keywords were divided into four clusters (Figure 7B). The latest keywords of the four clusters will describe future research hotspots in this field comprehensively and profoundly.

With respect to the latest research hotspots, in the cluster of functions of electroactive materials, the newest keyword of this cluster is a sensor with an AAY of 2018.9878. The potential reasons for this are as follows: First, electroactive materials are most commonly applied in sensors and actuators because of their ability to deliver electrical signals to cells and tissue (Bar-Cohen et al., 2017). Furthermore, because of the characteristics of sensors, electroactive materials are emerging as new disease-modifying therapies that offer the possibility of improving tissue repair and regeneration and re-establishing functionality at both the cellular and organ levels (Pinho et al., 2021). With developments in materials, manufacturing, biotechnology, and microelectromechanical systems (MEMS), many exciting biosensors and bioactuators have been developed based on biocompatible piezoelectric materials. These biodevices can be safely integrated into biological systems for applications such as sensing biological forces, stimulating tissue growth and healing, and diagnosing medical problems (Chorsi et al., 2019). This suggests that advances in sensors are laying the foundation for an entire field of development, and they are also becoming a hotspot in future research.

Regarding the topic of the characterisation of electroactive material clusters, the water contact angle was the most recent keyword, with an AAY of 2018.381. Biological interactions take place at the surface of biomaterials, where they are in direct contact with the host tissue. Some current studies confirm that one of the disadvantages of some biomaterials is their hydrophobicity, which limits their applications in TE. Moreover surfaces with a moderate wettability (water contact angle of 30–60°) have been shown to be favourable for cell adhesion and proliferation (Andrade et al., 1979; Hsu and Chen, 2000). Another study showed that electroactive barium titanate-coated titanium scaffolds improve osteogenesis and osseointegration for large segmental bone defects, and this success is closely related to the water contact angle (Fan et al., 2020). In view of this, Guo (Guo et al., 2012; Guo et al., 2019)^42,4342,43^ made electroactive porous tubular scaffolds with a hydrophilic surface with a water contact angle of approximately 30° by doping the films with (±)-10-camphorsulfonic acid. These degradable electroactive tubular scaffolds are good candidates for neural tissue engineering applications, and studies in this field are becoming more detailed (Guo et al., 2012). We predict that the hydrophilicity of electroactive materials in TE will be one of the evaluation standards for electroactive materials in the future. Therefore, determination of the water contact angle is an indispensable step in future electroactive material research. This also suggests that more attention should be paid to research on the water contact angles of similar materials.

Regarding the electroactive materials used in nerve and cardiac tissue engineering, the latest terms were silk fibroin (AAY 2018.3099) and conductive hydrogel (AAY 2018.662), occurring 71 times (Figure 7C). Nerve restoration and repair in the central nervous system are complicated and require several factors to be considered in the design of scaffolds, such as bioactivity and neuroinductive, neuroconductive, and antioxidant properties. According to publication numbers, the term silk fibroin (SF) has attracted more attention among researchers. SF has unique mechanical properties and biocompatibility (Altman et al., 2003), and nerve guidance channels made up of electrospun and woven silk fibroin/poly (lactic-co-glycolic acid) are biocompatible and have a favourable mechanical strength (Wang et al., 2015). In conservative therapy for myocardial infarction (MI), electroactive silk fibroin/PLA nanofibrous bioactive scaffolds have been proven to inspire the rejuvenation of injured myocardium (Yan et al., 2020). Similarly, cerium oxide nanoparticles encapsulated in fabricated hybrid silk fibroin nanofibres have been reported as an artificial neural guidance conduit applicable in peripheral nerve regeneration (Saremi et al., 2021). Although research on silk fibroin used in TE is still in its infancy, it provides new perspectives for therapeutic strategies in nerve tissue and cardiac tissue regeneration.

(Dvir et al., 2011) and (Shin et al., 2012; Khorshidi and Karkhaneh, 2018) reported a successful demonstration of a myocardial tissue engineering scaffold based on conductive hydrogel that can promote the bridging of electrical signal pathways of adjacent cells, thus achieving myocardial tissue regeneration and functional reconstruction. This success encouraged the development of a wide range of conductive hydrogel-based myocardial tissue engineering scaffolds. For example, Liu et al. (2022) and Wei et al. (2022) developed paintable and rapidly bondable conductive hydrogels as therapeutic cardiac patches, which can improve the reconstruction of cardiac function and revascularization of infarct myocardium (Wei et al., 2022). Works by Qian et al. (2018a), Qian et al. (2018b), and Xu et al. (2018) successfully demonstrated that nerve tissue engineering scaffold-coated graphene-loaded polycaprolactone based on a conductive hydrogel can transmit electrical signals, thus realising nerve tissue regeneration and functional reconstruction. This success has promoted the development of a wide range of nerve tissue-engineering scaffolds based on conductive hydrogels (Xu et al., 2018). Although conductive hydrogels have shown potential in tissue engineering applications, research on them is still in the early stages, because they currently have constraints, such as the inability to balance high mechanical properties and high electrical conductivity, and difficulties with adjusting mechanical properties (Liu et al., 2020). In practice, there is still abundant room for further progress in conductive hydrogel research on TE.

Regarding the cluster of applications of electroactive materials in bone tissue engineering, angiogenesis is the most recent keyword (cluster3), with an AAY of 2019.14. This word is also the latest keyword in all clusters. Recent research has shown that electroactive materials can induce vascular endothelial cell luminal formation *in vitro* and neovascularization *in vivo* (Augustine et al., 2017). Research indicated that polarised nanocomposite membranes and DBB granules have a synergistic effect in promoting bone defect repair by means of active early neovascularization (Bai et al., 2019). Meanwhile, in treatments for the regeneration of infectious bone defects, vancomycin—and strontium-loaded microspheres, which are made of a block copolymer consisting of poly (l-lactide) (PLLA) and poly (ethyl glycol) (PEG) blocks, have broad applications in the field of bone tissue engineering, one of which is its capacity for enhancing angiogenesis (Wei et al., 2019). Early neovascularization has a profound effect on subsequent bone remodelling and maturation; therefore, it has received increasing attention in recent years.

To summarise the general trends of the four groups in this study, the most fundamental one is the characterisation of electroactive materials. Recently, more attention has been paid to the application of electroactive materials in bone tissue engineering (Figure 7C), which suggests that although new functional materials are constantly being developed, attention should also be paid to their applications and subsequent transformations in tissue engineering. In addition, electroactive materials have some defects in application. First of all, some electroactive biomaterials may have immune rejection after implantation because of their poor biocompatibility. Secondly, some electroactive biomaterials have limited degradability, which limits their application as tissue regeneration and repair materials. Improving the above shortcomings will be the future direction of researchers.

In addition, in terms of research fields, subjects related to materials, such as physics, chemistry, and biology (Figure 8), are worthy of attention. In terms of research, research is not limited to material applications. Basial research, including molecular, mechanical, and immunological research (Figure 8), cannot be ignored. It is believed that the development of any field requires multidisciplinary communication and assistance, similar to what is currently seen for the electroactive biomaterials in the TE field.

### Limitation

Publications in the WOS database were investigated in this study to obtain objective and reliable results. It has been confirmed that document type labels in web of science are more accurate than Scopus in previous report, So we perform WOS-based retrieval preferentially (Yeung, 2019). However, owing to the limitation of searching for studies in English and constant updates of the database, our results may differ slightly from the reality. In addition, for more comprehensive results, databases such as Scopus, and Google Scholar could be included and compared in future studies. Due to the characteristics of database retrieval, we can’t analyse the hot spots and future trends of the application of electroactive materials in each type of tissue engineering in detail, and we hope to be able to more detailed analysis in the future research.

## Conclusion

China has contributed the most to the field of electroactive biomaterials used in TE, and Iran has shown the highest research interest in this area in recent years, and cooperation between countries is crucial. The number of future publications in this field is expected to increase. Sensors, the water contact angle, angiogenesis, silk fibroin, and conductive hydrogels are the focus of our attention in the future, and attention should also be paid to the applications and transformations of electroactive materials, and bone tissue engineering in particular. An overall analysis of the field from the perspective of physics, chemistry, biology, molecular mechanics, and immunology is the latest research direction. Similarly, the development of the field requires joint efforts from all disciplines.

## Data Availability

The original contributions presented in the study are included in the article/Supplementary Material, further inquiries can be directed to the corresponding authors.
