# Supplementary material for: Knowledge Domain and Hotspots Predict Concerning Electroactive Biomaterials Applied in Tissue Engineering: A Bibliometric and Visualized Analysis From 2011 to 2021
Source: Front Bioeng Biotechnol. 2022 May 23;10:904629. doi: 10.3389/fbioe.2022.904629 (PMC9168279; doi:10.3389/fbioe.2022.904629)
Supplement: Supplementary file 1 [file Table1.docx]

**Supplemental Table 1. The analytic consequence of 288 keywords with at least 35 occurrence times**

| **Label** | **Cluster** | **Occurrences** | **Average appearing years**  **(AAY)** | |
| --- | --- | --- | --- | --- |
| multiwalled carbon nanotube | 1 | 52 | | 2015.4423 |
| electrospun poly | 1 | 52 | | 2015.5962 |
| l lactide | 1 | 140 | | 2015.9143 |
| mwcnt | 1 | 101 | | 2016.1584 |
| mwcnts | 1 | 64 | | 2016.5 |
| afm | 1 | 38 | | 2016.6842 |
| atomic force microscopy | 1 | 37 | | 2016.7027 |
| dispersion | 1 | 101 | | 2016.7327 |
| blend | 1 | 85 | | 2016.7647 |
| cell morphology | 1 | 48 | | 2016.8125 |
| fibrous scaffold | 1 | 94 | | 2016.8191 |
| sbf | 1 | 43 | | 2016.9302 |
| epsilon caprolactone | 1 | 91 | | 2016.956 |
| diameter | 1 | 176 | | 2016.9716 |
| mtt | 1 | 47 | | 2016.9787 |
| electrospun scaffold | 1 | 67 | | 2017 |
| pore | 1 | 91 | | 2017.011 |
| poly | 1 | 810 | | 2017.0123 |
| differential scanning calorimetry | 1 | 39 | | 2017.0256 |
| high porosity | 1 | 36 | | 2017.0278 |
| electrospinning technique | 1 | 44 | | 2017.0682 |
| electrospun nanofiber | 1 | 71 | | 2017.0704 |
| body fluid | 1 | 79 | | 2017.0759 |
| microstructure | 1 | 89 | | 2017.1011 |
| degradation | 1 | 142 | | 2017.1127 |
| morphology | 1 | 435 | | 2017.1172 |
| elongation | 1 | 74 | | 2017.1216 |
| xps | 1 | 50 | | 2017.14 |
| dsc | 1 | 40 | | 2017.15 |
| nanofibrous scaffold | 1 | 177 | | 2017.1695 |
| x ray photoelectron spectroscopy | 1 | 49 | | 2017.1837 |
| electron microscopy | 1 | 190 | | 2017.1842 |
| nanofiber | 1 | 357 | | 2017.1989 |
| thermal stability | 1 | 62 | | 2017.2258 |
| fiber diameter | 1 | 70 | | 2017.2286 |
| copolymer | 1 | 75 | | 2017.24 |
| transmission electron microscopy | 1 | 60 | | 2017.25 |
| hap | 1 | 64 | | 2017.25 |
| mu m | 1 | 137 | | 2017.2555 |
| thermogravimetric analysis | 1 | 38 | | 2017.2632 |
| sem | 1 | 276 | | 2017.3152 |
| lactic acid | 1 | 63 | | 2017.3333 |
| electrospinning | 1 | 211 | | 2017.3365 |
| multi walled carbon nanotube | 1 | 58 | | 2017.3621 |
| assay | 1 | 181 | | 2017.3646 |
| x ray diffraction | 1 | 103 | | 2017.3689 |
| scanning electron microscopy | 1 | 103 | | 2017.3689 |
| dimethylthiazol | 1 | 35 | | 2017.3714 |
| surface roughness | 1 | 45 | | 2017.3778 |
| tem | 1 | 55 | | 2017.3818 |
| contact angle | 1 | 47 | | 2017.383 |
| decrease | 1 | 73 | | 2017.3836 |
| ft ir | 1 | 47 | | 2017.4043 |
| mtt assay | 1 | 83 | | 2017.4096 |
| characterization | 1 | 261 | | 2017.41 |
| hydroxyapatite | 1 | 130 | | 2017.4154 |
| increase | 1 | 279 | | 2017.4444 |
| modulus | 1 | 229 | | 2017.4454 |
| thermal property | 1 | 39 | | 2017.4615 |
| composite scaffold | 1 | 208 | | 2017.4663 |
| pore size | 1 | 110 | | 2017.5091 |
| cell line | 1 | 116 | | 2017.5259 |
| presence | 1 | 279 | | 2017.5269 |
| viability | 1 | 399 | | 2017.5363 |
| spectroscopy | 1 | 184 | | 2017.5435 |
| porous structure | 1 | 110 | | 2017.5545 |
| crystallinity | 1 | 59 | | 2017.5593 |
| tga | 1 | 47 | | 2017.5957 |
| young | 1 | 102 | | 2017.598 |
| raman spectroscopy | 1 | 53 | | 2017.6038 |
| compressive strength | 1 | 85 | | 2017.6235 |
| fourier | 1 | 153 | | 2017.6471 |
| bioactivity | 1 | 172 | | 2017.657 |
| cell attachment | 1 | 160 | | 2017.6813 |
| mpa | 1 | 129 | | 2017.7054 |
| water | 1 | 66 | | 2017.7121 |
| content | 1 | 261 | | 2017.7126 |
| porous scaffold | 1 | 104 | | 2017.7212 |
| degrees c | 1 | 68 | | 2017.7353 |
| porosity | 1 | 219 | | 2017.7443 |
| tensile strength | 1 | 136 | | 2017.7721 |
| elastic modulus | 1 | 63 | | 2017.7778 |
| pcl | 1 | 215 | | 2017.786 |
| polymer matrix | 1 | 59 | | 2017.7966 |
| toughness | 1 | 57 | | 2017.807 |
| functional group | 1 | 63 | | 2017.8095 |
| bone tissue engineering application | 1 | 129 | | 2017.8217 |
| surface morphology | 1 | 45 | | 2017.8222 |
| freeze | 1 | 75 | | 2017.8533 |
| wettability | 1 | 58 | | 2017.8966 |
| xrd | 1 | 108 | | 2017.8981 |
| nanocomposite scaffold | 1 | 74 | | 2017.9054 |
| degradation rate | 1 | 87 | | 2017.9425 |
| ftir | 1 | 132 | | 2017.9697 |
| pla | 1 | 75 | | 2017.9733 |
| degree | 1 | 115 | | 2017.9913 |
| fold | 1 | 58 | | 2018 |
| polycaprolactone | 1 | 133 | | 2018.0752 |
| sample | 1 | 165 | | 2018.1636 |
| hydrophilicity | 1 | 176 | | 2018.1761 |
| nanofiller | 1 | 49 | | 2018.3061 |
| pva | 1 | 56 | | 2018.375 |
| water contact angle | 1 | 42 | | 2018.381 |
| cell type | 2 | 68 | | 2016.6176 |
| nanotechnology | 2 | 75 | | 2016.7467 |
| cnt | 2 | 190 | | 2016.8053 |
| cnts | 2 | 141 | | 2016.8582 |
| stem cell differentiation | 2 | 55 | | 2017 |
| exposure | 2 | 69 | | 2017.1304 |
| great interest | 2 | 42 | | 2017.1429 |
| interest | 2 | 172 | | 2017.2209 |
| nanomedicine | 2 | 43 | | 2017.2558 |
| promise | 2 | 103 | | 2017.2621 |
| nanostructure | 2 | 95 | | 2017.3579 |
| use | 2 | 518 | | 2017.4054 |
| issue | 2 | 107 | | 2017.4112 |
| possibility | 2 | 73 | | 2017.4932 |
| delivery | 2 | 143 | | 2017.5175 |
| future | 2 | 60 | | 2017.5833 |
| advance | 2 | 102 | | 2017.5882 |
| engineering | 2 | 1366 | | 2017.5893 |
| functionalization | 2 | 130 | | 2017.6 |
| organ | 2 | 85 | | 2017.6353 |
| generation | 2 | 104 | | 2017.6442 |
| unique property | 2 | 63 | | 2017.6508 |
| system | 2 | 388 | | 2017.6804 |
| biosensor | 2 | 98 | | 2017.6837 |
| biomedicine | 2 | 67 | | 2017.6866 |
| perspective | 2 | 94 | | 2017.7128 |
| application | 2 | 1513 | | 2017.729 |
| chemical property | 2 | 67 | | 2017.7313 |
| form | 2 | 116 | | 2017.7414 |
| variety | 2 | 112 | | 2017.7589 |
| research | 2 | 293 | | 2017.7679 |
| area | 2 | 311 | | 2017.7685 |
| development | 2 | 539 | | 2017.7755 |
| platform | 2 | 230 | | 2017.7783 |
| opportunity | 2 | 72 | | 2017.7917 |
| mechanism | 2 | 208 | | 2017.8173 |
| body | 2 | 84 | | 2017.8214 |
| year | 2 | 52 | | 2017.8462 |
| biomolecule | 2 | 65 | | 2017.8462 |
| wide range | 2 | 84 | | 2017.8571 |
| importance | 2 | 86 | | 2017.8605 |
| need | 2 | 99 | | 2017.8687 |
| therapy | 2 | 240 | | 2017.9 |
| drug | 2 | 123 | | 2017.9106 |
| derivative | 2 | 115 | | 2017.9304 |
| field | 2 | 490 | | 2017.9429 |
| graphene | 2 | 426 | | 2017.9437 |
| regenerative medicine | 2 | 342 | | 2017.9532 |
| detail | 2 | 53 | | 2017.9623 |
| toxicity | 2 | 151 | | 2017.9735 |
| efficacy | 2 | 81 | | 2017.9753 |
| state | 2 | 105 | | 2017.981 |
| biomedical application | 2 | 319 | | 2017.9812 |
| direction | 2 | 112 | | 2018 |
| nanomaterial | 2 | 333 | | 2018.036 |
| example | 2 | 60 | | 2018.0667 |
| advantage | 2 | 158 | | 2018.0886 |
| strategy | 2 | 400 | | 2018.09 |
| drug delivery | 2 | 220 | | 2018.0909 |
| focus | 2 | 62 | | 2018.0968 |
| biomedical field | 2 | 78 | | 2018.1026 |
| progress | 2 | 126 | | 2018.1032 |
| researcher | 2 | 54 | | 2018.1111 |
| device | 2 | 187 | | 2018.1444 |
| medicine | 2 | 88 | | 2018.1477 |
| context | 2 | 46 | | 2018.1522 |
| article | 2 | 95 | | 2018.1895 |
| integration | 2 | 100 | | 2018.2 |
| disease | 2 | 165 | | 2018.2061 |
| carbon | 2 | 125 | | 2018.216 |
| clinical application | 2 | 70 | | 2018.2286 |
| drug delivery system | 2 | 58 | | 2018.2414 |
| limitation | 2 | 99 | | 2018.2525 |
| attention | 2 | 230 | | 2018.2957 |
| technology | 2 | 191 | | 2018.2984 |
| recent year | 2 | 79 | | 2018.3038 |
| review | 2 | 440 | | 2018.3045 |
| biosensing | 2 | 52 | | 2018.3077 |
| patient | 2 | 83 | | 2018.3494 |
| regard | 2 | 44 | | 2018.3864 |
| functionality | 2 | 142 | | 2018.4366 |
| challenge | 2 | 361 | | 2018.4515 |
| light | 2 | 55 | | 2018.4727 |
| overview | 2 | 82 | | 2018.4878 |
| cancer therapy | 2 | 39 | | 2018.5385 |
| skin | 2 | 59 | | 2018.678 |
| physicochemical property | 2 | 87 | | 2018.6782 |
| recent advance | 2 | 92 | | 2018.6957 |
| biomedical applications | 2 | 40 | | 2018.8 |
| healing | 2 | 167 | | 2018.8204 |
| metal | 2 | 45 | | 2018.8667 |
| sensor | 2 | 82 | | 2018.9878 |
| plla scaffold | 3 | 112 | | 2015.75 |
| hmsc | 3 | 76 | | 2016.1711 |
| lactic co glycolic acid | 3 | 54 | | 2016.4259 |
| plla | 3 | 461 | | 2016.4989 |
| l lactic acid | 3 | 218 | | 2016.5138 |
| human mesenchymal stem cell | 3 | 80 | | 2016.525 |
| osteoblast | 3 | 149 | | 2016.5973 |
| plga | 3 | 88 | | 2016.6023 |
| bone marrow | 3 | 84 | | 2016.8333 |
| msc | 3 | 110 | | 2016.8455 |
| mineralization | 3 | 131 | | 2016.8473 |
| week | 3 | 138 | | 2016.8623 |
| alkaline phosphatase | 3 | 96 | | 2017.0104 |
| mscs | 3 | 72 | | 2017.0556 |
| stem cell | 3 | 336 | | 2017.1458 |
| day | 3 | 322 | | 2017.2143 |
| alp | 3 | 93 | | 2017.2473 |
| mc3t3 e1 cell | 3 | 44 | | 2017.2727 |
| deposition | 3 | 165 | | 2017.2909 |
| present study | 3 | 131 | | 2017.3359 |
| alkaline phosphatase activity | 3 | 75 | | 2017.4 |
| poly l lactic acid | 3 | 95 | | 2017.4737 |
| col | 3 | 44 | | 2017.5 |
| growth factor | 3 | 123 | | 2017.5041 |
| gene | 3 | 159 | | 2017.522 |
| expression | 3 | 290 | | 2017.5241 |
| gene expression | 3 | 105 | | 2017.5429 |
| bone tissue engineering | 3 | 513 | | 2017.5439 |
| bone formation | 3 | 86 | | 2017.5581 |
| control group | 3 | 47 | | 2017.5745 |
| rat | 3 | 110 | | 2017.5818 |
| osteogenic differentiation | 3 | 230 | | 2017.6 |
| bone repair | 3 | 56 | | 2017.6071 |
| human adipose | 3 | 39 | | 2017.6154 |
| significance | 3 | 57 | | 2017.6316 |
| mesenchymal stem cell | 3 | 284 | | 2017.6585 |
| cartilage tissue engineering | 3 | 50 | | 2017.68 |
| bone tissue regeneration | 3 | 69 | | 2017.7246 |
| regulation | 3 | 56 | | 2017.7857 |
| alp activity | 3 | 44 | | 2017.7955 |
| defect | 3 | 158 | | 2017.8291 |
| bone morphogenetic protein | 3 | 47 | | 2017.8511 |
| pathway | 3 | 99 | | 2018.0202 |
| runx2 | 3 | 39 | | 2018.0513 |
| endothelial cell | 3 | 73 | | 2018.0685 |
| statement | 3 | 52 | | 2018.0962 |
| bone regeneration | 3 | 226 | | 2018.1062 |
| vivo study | 3 | 54 | | 2018.1111 |
| bmp | 3 | 45 | | 2018.1111 |
| bone tissue | 3 | 88 | | 2018.1818 |
| osteogenesis | 3 | 132 | | 2018.447 |
| synergistic effect | 3 | 80 | | 2018.45 |
| bmsc | 3 | 60 | | 2018.45 |
| bone defect | 3 | 118 | | 2018.6271 |
| promising strategy | 3 | 43 | | 2018.6744 |
| angiogenesis | 3 | 50 | | 2019.14 |
| neurite outgrowth | 4 | 46 | | 2015.9783 |
| pedot | 4 | 40 | | 2017.1 |
| electrical signal | 4 | 42 | | 2017.119 |
| pluripotent stem cell | 4 | 44 | | 2017.3409 |
| pani | 4 | 75 | | 2017.36 |
| polyaniline | 4 | 101 | | 2017.4554 |
| alignment | 4 | 92 | | 2017.5326 |
| neuron | 4 | 103 | | 2017.534 |
| polypyrrole | 4 | 119 | | 2017.5462 |
| polymerization | 4 | 119 | | 2017.563 |
| peripheral nerve regeneration | 4 | 40 | | 2017.6 |
| ppy | 4 | 94 | | 2017.6064 |
| nerve tissue engineering | 4 | 75 | | 2017.6133 |
| electrical property | 4 | 99 | | 2017.6263 |
| neural stem cell | 4 | 56 | | 2017.6786 |
| electrical stimulation | 4 | 202 | | 2017.7178 |
| schwann cell | 4 | 44 | | 2017.7273 |
| neural tissue engineering | 4 | 104 | | 2017.7596 |
| s cm | 4 | 67 | | 2017.7761 |
| maturation | 4 | 74 | | 2017.8243 |
| cardiomyocyte | 4 | 95 | | 2017.8421 |
| conductive scaffold | 4 | 89 | | 2017.8764 |
| conductive polymer | 4 | 93 | | 2017.9032 |
| conductivity | 4 | 473 | | 2017.9218 |
| myocardial infarction | 4 | 47 | | 2017.9362 |
| nerve regeneration | 4 | 79 | | 2017.9494 |
| stimulation | 4 | 139 | | 2017.9712 |
| conductive material | 4 | 46 | | 2017.9783 |
| cardiac tissue engineering | 4 | 93 | | 2018.0108 |
| microenvironment | 4 | 167 | | 2018.012 |
| ethylenedioxythiophene | 4 | 56 | | 2018.0357 |
| cardiac tissue | 4 | 53 | | 2018.1321 |
| heart | 4 | 55 | | 2018.1636 |
| injury | 4 | 152 | | 2018.2039 |
| nerve | 4 | 61 | | 2018.2131 |
| silk fibroin | 4 | 71 | | 2018.3099 |
| conductive hydrogel | 4 | 71 | | 2018.662 |
